# The four causes: the functional architecture of centromeres and kinetochores

**DOI:** 10.1146/annurev-genet-072820-034559

**Published:** 2022-09-02

**Authors:** Andrew D. McAinsh, Adele L. Marston

**Affiliations:** 1 Centre for Mechanochemical Cell Biology, University of Warwick, Coventry CV4 7AL, UK; 2 Division of Biomedical Sciences, Warwick Medical School, University of Warwick, Coventry CV4 7AL, UK; 3 The Wellcome Centre for Cell Biology, Institute of Cell Biology, School of Biological Sciences, Michael Swann Building, Max Born Crescent, Edinburgh, EH9 3BF UK

**Keywords:** kinetochore, centromere, mitosis, meiosis, microtubule, chromosome, cell division, mitotic spindle, chromatin

## Abstract

Kinetochores are molecular machines that power chromosome segregation during the mitotic and meiotic cell divisions of all eukaryotes. Aristotle explains how we think we have knowledge of a thing only when we have grasped its cause. The four causes correspond to questions that one must ask to gain understanding of natural phenomena - which in our case is the Kinetochore: 1) What are the constituent parts? 2) How does it assemble? 3) What is the structure and arrangement? and 4) What is the function? Here we outline the current blueprint for the assembly of a kinetochore, how functions are mapped onto this architecture and how this is shaped by the underlying pericentromeric chromatin. This is possible because an almost complete parts list of the kinetochore is now available alongside recent advances using *in vitro* reconstitution, structural biology and genomics. In many organisms each kinetochore binds to multiple microtubules and we propose a model for how this ensemble-level architecture is organised, drawing on key insights from the simple “one microtubule-one kinetochore” setup in budding yeast and innovations that enable meiotic chromosome segregation.

## 1 Introduction

**Figure 1:**
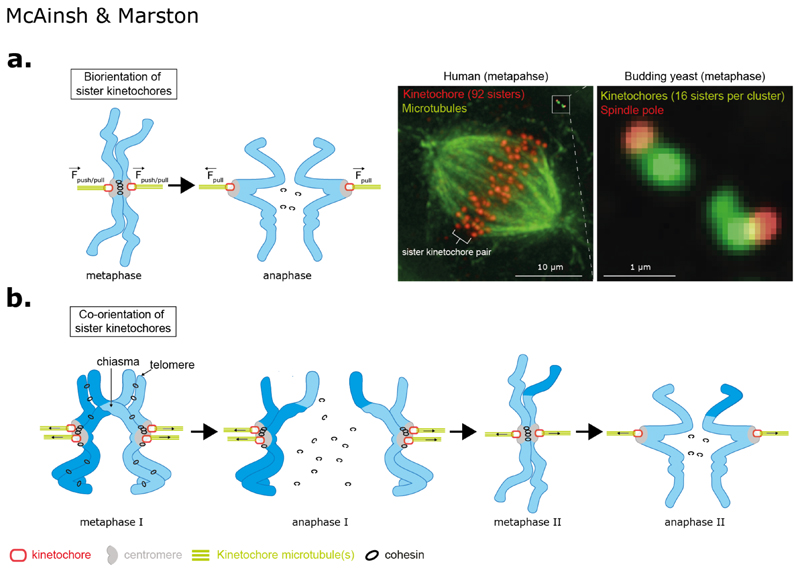
Geometries of chromosome segregation during mitosis and meiosis. **(a)** *Top left:* In mitosis the replicated chromosomes (sister chromatids - blue) are bioriented with sister kinetochores (red) in a back-to-back geometry and embedded in pericentromeric chromatin domain (grey). Sister chromatids are physically held together by cohesin molecules which trap the two DNA stands (black circles). The plus-end of spindle microtubules (green; either singular in budding yeast or multiple in animal cells) are embedded in the kinetochore with their minus-ends projecting towards the centrosomes (human) or spindle pole bodies (budding yeast). Pulling forces generated by kinetochore microtubule attachments pull sister chromatids apart in anaphase once cohesin is cleaved (on satisfaction of the spindle assembly checkpoint). *Top Right:* Mitotic spindle in a human cell (kinetochores red and microtubules (green) compared to budding yeast (kinetochores green and spindle pole bodies in red). In yeast the 32 sister kinetochores form two clusters along the spindle axis, which is ~1 μm in length. This is similar to distance between two sister kinetochores in humans. In humans the sister kinetochores are aligned along the spindle axis. **(b)** In meiosis I, replicated maternal and paternal (homologous) chromosomes are physically connected are a result of crossover recombination, which generates chiasmata, together with sister chromatid cohesion distal to the chiasmata. Sister kinetochores are attached to microtubules from the same pole and are said to be co-oriented. An anaphase I, cohesin is cleaved only on chromosomes arms (pericentromeric cohesin is protected from cleavage by shugoshin-PP2A; reviewed in (158)) which resolves chiasma and allows homologous chromosomes to segregate to opposite poles. In meiosis II, sister kinetochores biorient and the pericentromeric cohesin resists the pulling forces from microtubules. During anaphase II, pericentromeric cohesin is cleaved and sister kinetochores segregate to opposite poles.

Kinetochores are macro-molecular protein assemblies, the canonical function of which is to form load-bearing attachments to the plus ends of spindle microtubules on eukaryotic chromosomes. In mitosis, the identical sister chromatids, which are held together by cohesin, attach via their sister kinetochores to microtubules from opposite spindle poles (Fig. 1a). Kinetochores, together with cohesin, provide resistance and coupling to spindle microtubule-derived forces, generating tension and chromosome movement. Once this state of sister kinetochore biorientation has been achieved for all sister chromatid pairs, cohesin is abruptly lost, resulting in the equational segregation of sister chromatids. While not the focus of this review, these events are tightly regulated by 1) the spindle assembly checkpoint (SAC) which prevents anaphase onset when one or more kinetochores are unattached (140) and 2) error correction mechanisms that destabilise erroneous attachments and promote bi-orientation (138). These are key during early mitosis when kinetochores are in a mixture of attachment states. For example, in budding yeast attachments are initially syntelic with error-correction driving bi-orientation and detachment is rare, explaining why the SAC is non-essential (156). On the other hand, in animal cells the default state is unattached and SAC active. Here, kinetochores need to first capture microtubules through either side-on or end-on interactions giving rise to proper (amphitelic) as well as improper (syntelic or merotelic) attachments that require correction. Remarkably, these processes are then adapted during meiosis, where sister kinetochores attach to microtubules from the same pole (co-orientation) during the first division so that sister chromatids co-segregate to allow for a reduction in ploidy (Fig. 1b).

To enable these multiple functions, kinetochores are built from multiple copies of multiple proteins and complexes which, although not highly conserved at the sequence level, have recognisable homologs and adopt a similar architecture in most studied eukaryotes, with some variations (167, 240). A notable exception are kinetoplastids, which have divergent kinetochores with distinct protein origins, and some insects where kinetochores form a layer across the whole chromosome (108, 241). In contrast, centromeres, the chromatin loci where kinetochores assemble, are highly divergent and rapidly evolving. In their simplest form, as in the budding yeast *Saccharomyces cerevisiae*, centromeres are defined by a specific ~125bp DNA sequence, which is more-or-less the same on all 16 chromosomes and referred to as a point centromere (161). However, in most eukaryotes, centromeres are not defined by sequence and consist of highly repetitive DNA sequences such as tandem repeats and retrotransposons that are unrelated in different organisms and vary even between chromosomes of the same organism. These complex centromeres are known as regional centromeres and can extend for several megabases (5). In humans, for example, many centromeres are composed of so-called alpha satellite repeats (237). Budding yeast centromeres wrap a single centromeric (CenpA-containing) nucleosome and each kinetochore binds a single microtubule (74, 260). Regional centromeres contain many CenpA nucleosomes and assemble compound kinetochores that bind multiple microtubules (10-15 in human) (46, 122, 181, 210). Each centromere/kinetochore is flanked by a specialized chromosomal domain, called the pericentromere. In most organisms, pericentromeres are large, extending from several kb (fission yeast) to megabases (humans), repetitive, heterochromatic and cohesin-rich. In budding yeast, pericentromeres are compact (~20kb) and lack heterochromatin but are nevertheless highly enriched with cohesin (157). Kinetochore structure and function must therefore be considered in the context of a specialized chromatin environment.

## 2 Kinetochore assemblage

**Figure 2:**
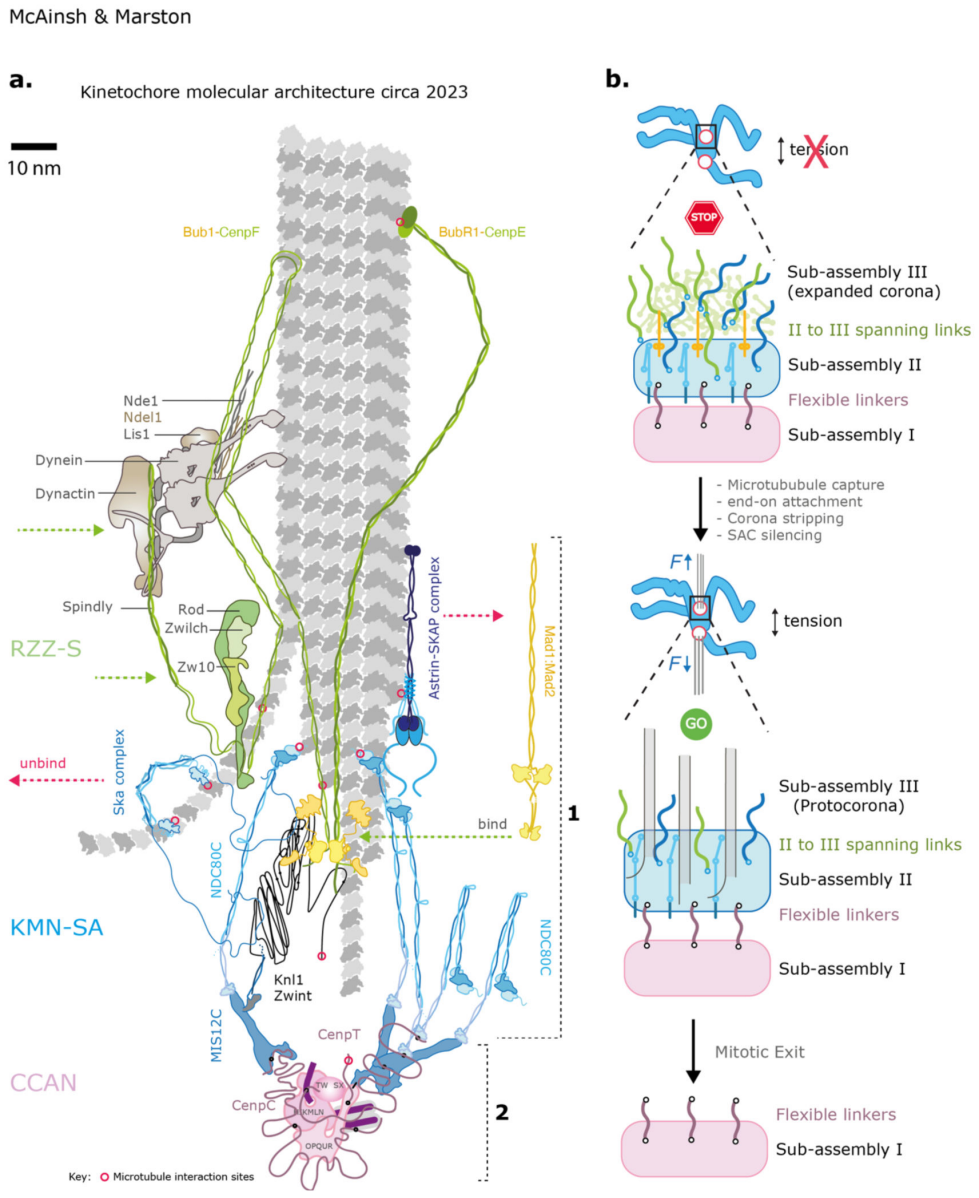
Molecular architecture of the kinetochore **(a)** Architecture of a single microtubule-kinetochore attachment site. For clarity only one CCAN (pink) and the associated molecules is shown (see Fig. 3 for extension of models to multi-subunit kinetochores). All molecules are drawn to scale based on known structural biology, length of coiled-coil sequences or length of disordered regions. The relative position of molecules is informed by the measured Euclidian distances between the average positions of two labelled proteins in a population of kinetochores (see (213)) and/or known binding interfaces. Key features: Red circles donate known contact points between a protein and the microtubule. Flexible linkers connect CCAN to KMN (**1**) extended coiled-coil elements span subassembly II to sub-assembly III (CenpF, CenpE, Mad1). Detachment of microtubules triggers a switch in composition and architecture: SAC factors (yellow) including Mad1:Mad2 load on Bub1-Bub3 that are bound on the Knl1 phospho-domain (black dots) which causes rearrangement of NDC80C as they jack-knife and loose order (**2**). Other factors that load or leave are designated by green and red dotted arrows respectively. Not all factors are shown. Scale bar = 10 nm. **(b)** Dynamic remodelling of kinetochores: at the start of mitosis kinetochores have not yet established amphitelic attachment and the SAC (yellow molecule) is actively delaying anaphase onset. In humans, there is expansion of subassembly III (green) into the corona founded on self-assembly of RZZ (light green). As end on attachments form, the corona (and SAC) is disassembled in part by dynein-driven stripping of corona cargoes. This leaves residual corona molecules spanning to subassembly II. Stretching of linkers separates subassembly I (pink) and II (blue ) when under tension while there are conformational changes within the latter.

Conventional kinetochores consist of ~100 proteins (see Table 1), many of which are organised into distinct complexes, that self-assembly in a hierarchical manner onto a specialized nucleosome. Fig. 2a sketches out the architecture of the core attachment site (approximate to scale). We will use the examples of budding yeast and human kinetochores to introduce these sub-complexes and how they can be assigned to three major sub-assemblies. Moving from centromeric DNA to microtubules, the sub-assemblies are: (*i)* specialized Cenp-A nucleosomes, *(ii)* CCAN: constitutive centromere associated network (also called Ctf19 complex in budding yeast), *(iii)* KMN-S network: incorporates Knl1, Mis12, Ndc80 and Ska complexes (or unrelated Dam1 complex in yeast that couples kinetochore to microtubules), which provides the core microtubule-binding interface and platform for spindle assembly checkpoint (SAC) and error correction processes, *(iv)* Corona (not in yeast): incorporates the Rod-Zw10-Zwilch-Spindly (RZZ-S), CenpF-Nde1-Ndel1-Lis1 (FNNL) complexes and molecular motors Dynein-Dynactin, CenpE and Kif2b.This facilitates microtubule capture, transport and SAC activities. This assemblage is not static but undergoes dynamic remodelling throughout the cell cycle (Fig 2b). Sub-assembly *(iv)* is a feature of unattached kinetochores, while in animal cells sub-assembly *(iii)* loads in early mitosis, undergoes a maturation process as microtubule attachments from, and disassembles in late anaphase (93). Sub-assembly *(i)* is present throughout the cell cycle, although there are hints of changes in organisation (6, 21, 162). The stoichiometry, stability and conformational state of sub-assemblies is clearly subject to dynamic change in response to mechanical (microtubule attachment and tension) and regulatory inputs i.e. cycles of phosphorylation driven by the major mitotic kinases (Cdk1, Aurora B, Mps1, Bub1, Haspin and Plk1) and phosphatases (PP1/PP2A)(114, 219). We will discuss these sub-assemblies in turn, highlighting key regulatory and functional features:

### 3.1 Sub assembly I (CenpA chromatin – a specialized nucleosome specifying centromere identity)

Kinetochore assembly must be restricted to a single site to avoid chromosome breakages due to opposing microtubule attachments in mitosis. In most organisms, the site of kinetochore assembly is defined by specialized nucleosomes in which the histone H3 subunit is replaced by the CenpA variant. Understanding how CenpA nucleosomes are specifically deposited at centromeres and how they are specifically recognised by the building blocks of the kinetochore are key questions in understanding centromere identity (for review see (166)).

Budding yeast “point” centromeres consist of three centromere determining elements (CDEs). *CDEI* (8bp) binds the helix-loop-helix transcription factor Cbf1, *CDEII* (80-90bp AT-rich sequence) wraps a single CenpA nucleosome and *CDEIII* (~25bp) binds the four subunit CBF3 complex (for review see (19). CBF3 comprises one copy each of Ndc10 and a CBF3core (Skp1, Ctf13 and two copies of Cep3). CBF3 binds the essential CCG and TCT motifs of CDEIII through the Gal4 domain of one of the Cep3 protomers, in a manner resembling transcription factor-promoter interactions (88, 142, 265, 272). CBF3 interacts with *CEN* DNA as a head-to-head dimer that includes CDEIII, leaving space for wrapping a Cse4-nucleosome with CDEII DNA (265). Recent data proposes an alternative view in which interactions between CBF3core and the nucleosome facilitate a hand-over from CBF3 to Cse4-nucleosomes at the *CEN* DNA (88). The Ndc10 subunit of CBF3 also recruits Scm3, a specific chaperone for Cse4 (budding yeast CenpA). This defines the deposition and placement of the CenpA nucleosome, though the exact mechanism is unclear (41, 56, 88, 173, 277).

Most human “regional” centromeres contain repeating units of two alternating 171bp a-satellite DNA sequences, one of which contains a 17bp “CenpB” box to which the CenpB protein binds in a sequence-specific manner. However, unlike in budding yeast, DNA sequence is not sufficient to dictate centromere assembly in humans and CenpB is not essential, although increasing numbers of these elements biases chromosomes towards faithful segregation (63). Instead, human centromeres, like those of most studied species, are defined epigenetically. At regional centromeres, existing CenpA directs assembly of new CenpA through an epigenetic loop. CenpC, a structural kinetochore component which directly binds the CenpA nucleosome, recruits the Mis18 complex, which in turn binds the HJURP chaperone (equivalent of Scm3 in yeast) to promote CenpA deposition. This process is tightly temporally regulated so that CenpA deposition occurs only at mitotic exit and during G1. During S phase, CenpA nucleosomes distribute onto the two nascent strands and it is in this concentration that they will provide the blueprint for kinetochore assembly and chromosome segregation. It is thought that upon CenpA dilution at S phase, canonical H3-containing nucleosomes act as placeholders that are evicted by transcription at mitotic exit. Centromere specification and CenpA deposition have been discussed in some excellent recent reviews (55, 166, 258).

Three key features of CenpA nucleosomes distinguish them from H3 nucleosomes and are important for defining centromere identity. First, the CenpA centromere-targeting domain (CATD), which is the region with the highest sequence divergence from H3, and sufficient for binding Scm3/HJURP, is critical for CenpA deposition at centromeres (71). Second, partly as a result of increased hydrophobicity of its C-terminal tail, CenpA confers preferential binding of CenpC which provides the base for kinetochore assembly (120). Third, CenpA nucleosomes differ from H3 nucleosomes in that they wrap less DNA (~100-120bp rather than 146bp) and the terminal DNA is less tightly bound, which has important implications for recruitment of the CCAN kinetochore sub-complex ((48, 67); see below).

### 3.2 Sub-assembly II (CCAN)

Human CCAN is made up of 16 proteins organised into 5 sub-complexes plus CenpC (72, 109, 184) The related Ctf19 complex (CTF19C) similarly has 5 sub-complexes in addition to CenpC/Mif2 made up of 14 proteins, the majority of which are recognisable orthologs of the human CCAN proteins (Table 1). Low sequence conservation and disparities in phenotype caused by loss of CCAN/CTF19C subunits had questioned the level of conservation of the yeast and human complexes. However, recent structural analyses of individual sub-complexes and the complete CCAN complex from yeast and human has revealed remarkable structural conservation of the entire complex (97–99, 192, 193, 248, 256, 264, 266, 273). Crucially, reconstitution and cryo-EM of both CTF19C and CCAN bound to CenpA nucleosomes indicate highly similar modes of binding (264, 266).

CCAN is built upon CenpC (Mif2) which has been termed the “blueprint” of the kinetochore (128). CenpC binds directly to the CenpA nucleosome and, despite being largely disordered, provides the structural platform upon which the kinetochore is assembled (128, 169, 201). The nucleosome-recognition and kinetochore assembly functions are conferred by separate linear binding motifs. The N-terminal region of human CenpC, which contains a Mis12-interacting domain followed by motifs that interact directly with CCAN sub-complexes CENP-LN and CENP-HIKM, templates kinetochore assembly (128, 190, 193, 226, 266). Two related “central” and “CenpC” regions, each comprised of a stretch of positively charged residues followed by two aromatic residues, bind to the acidic patch and C-terminal tail, respectively, on the CenpA nucleosome to specify the site of kinetochore assembly (32, 120). Either one of the central or CenpC domains appear to be sufficient for centromere targeting (250). Finally, the C-terminal region of CenpC dimerizes through its structured cupin domain, which at least *in vitro*, allows it to bind two nucleosomes, though the significance of this for kinetochore function *in vivo* is unclear (165, 248).

Although not highly conserved at the sequence level, the overall organisation of budding yeast CenpC/Mif2 is similar to that of human CenpC. In addition to connecting to the outer kinetochore through the Mis12 binding motif in its N-terminus, budding yeast CenpC also binds CCAN, although this was found to involve the CENP-QU (Okp1-Ame1) subunits rather than CENP-LN and CENP-HIKM as reported for human CenpC (58, 104). Resolving whether these observations underlie structural differences between the budding and human kinetochores or different kinetochore assembly states awaits a complete picture of a fully assembled kinetochore in both systems. In particular, the intrinsic disorder of CenpC has made structural analysis challenging. A further notable difference is that yeast CenpC, in common with other non-mammals, lacks the central CenpC domain so that CenpA/Cse4 recognition occurs solely through the CenpC domain (47, 262).

Recent cryo-EM structures have shown that human CCAN sub-complexes represent structural modules with CENP-OPQUR and CENP-HIKM forming two “lobes” or “pillars” bound to either side of the arc-shaped CENP-LN module. CENP-TW forms a base, connecting the two pillars and creating a positively-charged CENP-LN central channel (193, 266). In cryo-EM structures of the CenpA nucleosome bound to CCAN, this CENP-LN channel grips a-satellite linker DNA (266). These protein-DNA contacts appear to represent the major interaction surfaces between the assembled CCAN and CenpA supercomplex, apart from a small protein-protein interaction between CenpL and CenpA, together with the interactions between CenpC and CenpA nucleosome described above (266). Interestingly, the CENP-TWSX sub-complex which includes four histone fold domains, and is structurally related to the H3-H4 tetramer, wraps DNA as it emerges from the CENP-LN channel, introducing curvature into the DNA which threads through a groove supported by also by CenpI (266).

Budding yeast CTF19C has a remarkably similar architecture to human CCAN although it forms a shallower, wider channel. This is in part due to the absence of CenpM, which is sandwiched between the two pillars of human CCAN, deepening the channel (98, 193, 264, 266). Unwrapped nucleosome DNA, rather than linker DNA, was observed to be gripped by the CTF19C channel (264). It is also unclear whether DNA is topologically entrapped by CTF19C since CENP-TW (Cnn1-Wip1) was not clearly resolved, though modelling indicates that they have the potential to close the channel (98, 99, 264). The yeast homologs of CenpS and CenpX (Mhf1 and Mhf2) do not appear to be kinetochore proteins (139). Budding yeast also lack CenpR but this is substituted by Nkp1/Nkp2, forming a cap at the top of pillar 1 (193, 266). There is also evidence of functional divergence in human CCAN: the CENP-OPQUR subcomplex is a central receptor for polo-like kinase (Plk1) 1 working alongside Bub1 (15, 118, 177, 228), and displays Ndc80-like microtubule binding activity via an N-terminal extension that is absent in budding yeast (192).

The complete CTF19C/CCAN structures are largely consistent with prior studies addressing the arrangement and interaction with the CenpA nucleosome, with one major exception. Isolated vertebrate CenpN binds directly to the L1 loop of nucleosomal CenpA, an interaction that is thought to be important for specifying the site of kinetochore assembly (32, 33, 40, 91, 190). However, in the context of the complete CCAN, CenpN binding to CenpA L1 loop would cause a major steric clash. If the architecture of the complete CCAN-CenpA-nucleosome structures represents that of a fully assembled kinetochore, it is reasonable to assume that CenpN binding to the L1 loop of CenpA is an important assembly intermediate. Similarly, the Ame1-Okp1 (CENP-QU) heterodimer binds to the unmodified Cse4 (CenpA) N terminal tail in budding yeast and Cse4 and Ame1-Okp1 have been found in proximity by cross-linking mass spectrometry (9, 70). Whether this interaction is indicative of an assembly intermediate or representative of a full kinetochore assembly remains unclear.

#### 3.2.1 Impact of CCAN subunit disruption

In budding yeast, three CCAN subunits (Okp1/Ame1/Mif2) are encoded on essential genes while the remainder are indispensable for viability - albeit associated with increased frequency of chromosome mis-segregation (54, 68, 164, 185, 200). The picture in humans is complicated since: (1) results from acute or chronic knockdown/out experiments can vary with regard to the penetrance of chromosome alignment phenotypes; and (2) there is emerging evidence of cell type specific requirements (72, 163, 177, 192, 202). Differences in essentiality may also underlie the different extent to which different organisms rely on functional modules linking the centromeric nucleosome to the microtubule *i.e.* there are a few different molecular paths which involve different interactions between the CenpC and KMN which are not as used in some organisms compared to others (see below for details).

### 3.3 Sub-assembly III (KMN+SA)

The outer kinetochore is built from the KNL1, MIS12, NDC80 and SKA complexes, which have distinct functions. The KNL1 complex is an assembly hub for regulators that signal the attachment state of the kinetochore. The MIS12 complex connects the inner and outer kinetochore. The NDC80 and SKA complexes form the main microtubule-binding interface of the kinetochore, with the latter the major microtubule receptor (259). All are essential genes in yeast with protein inactivation in humans impacting chromosome alignment (to varying extents; comments above on CCAN being relevant).

#### The MIS12 complex

assembles from four structural paralogs, Dsn1, Mis12, Nsl1 and Pmf1 (Dsn1-Mtw1-Nsl1-Nnf1 in yeast) which bundle in parallel to form an elongated 20 nm long Y shape (58, 103, 159, 196, 198). The N terminal regions of Dsn1-Nsl1 and Mis12-Pmf1, respectively, form the tips of the Y and connect to the inner kinetochore through a direct interaction of Mis12 with CenpC (226). The stalk of the Y links to both the NDC80 and KNL1 complexes (197).

#### The NDC80 complex

is a 62 nm dumbbell-shaped heterotetramer formed from the Ndc80/Hec1:Nuf2; and Spc24:Spc25 dimers. Each dimer forms an N-terminal globular domain and a coiled-coil stalk. The coiled-coil C-termini of the two dimers intercalate in a tetrameric junction to assemble the NDC80C. A break in the Nuf2:Ndc80 coiled-coil forms a loop that is reported to interact with other various kinetochore/microtubule proteins depending on the organism (236) and provide rotational freedom. In the absence of microtubules, NDC80C jack-knifes into an autoinhibited state (220). In cells, this jack-knifed state correlates with SAC activation (Mad1:Mad2 binding) and may function as a microtubule occupancy sensor (10, 213, 249) (Fig. 2a, **step 1**). At the centromere facing end, Spc24:Spc25 form RWD domains that bind MIS12 complex or CenpT, as part of two distinct connections between the inner kinetochore and microtubules (see below). At the other end, Nuf2 and Ndc80 form calponin homology (CH) domains which form the main microtubule-binding interface of the kinetochore (37, 42, 43, 107, 242, 253–255). Part of the Ndc80 CH domain known as the “toe”, interacts directly with the microtubule lattice, binding at both the interface between alpha and beta tubulin monomers and at the interface between alpha-beta tubulin dimers (7, 8). The disordered, positively charged, N terminal “tail” of Ndc80, which has been extensively studied, also contributes to microtubule attachment and this is negatively regulated by phosphorylation (see error correction below; reviewed in (259)). NDC80 complexes bind microtubule lattices with low affinity and prefer straight versus curved protofilaments (37). *In vitro* experiments show that clusters of two or more NDC80 complexes can track with depolymerizing microtubules, and can stall and rescue microtubule depolymerization in a force-dependent manner (247).

#### The KNL1 complex

heterodimer of Knl1 and Zwint (known as Spc105 and Kre28 in budding yeast). A region towards the C-terminus of Knl1, that is predicted to form a coiled-coil, binds Zwint and is followed by tandem RWD domains which bind the stalk of Mis12 and a C-terminal motif in Nsl1 (197). The remainder of Knl1 is a large disordered element ((81); predicted ~400 nm in human) containing a series of motifs which bind key kinetochore regulators to provide an assembly platform for signalling the attachment state of the kinetochore. These motifs include a binding site for the PP1 phosphatase close to the N-terminus and multiple MELT motifs which, upon phosphorylation by the Mps1 kinase, dock a complex of the Bub1-Bub3 spindle assembly checkpoint (SAC) proteins which facilitates recruitment of Bub3-BubR1-PP2A (81, 132). Bub1:Bub3 also recruits Mad1:Mad2 complexes that catalyse the generation of a “wait anaphase signal” (reviewed in (140) and references therein). A series of feedback and forward loops between Mps1/AuroraB kinases and PP1/PP2A regulate the stability of attachments and promote checkpoint silencing (219).

#### SKA Complex

Metazoans contain an additional outer sub-assembly component. Ska1, Ska2 and Ska3 form a trimer that dimerises to from a W-shaped complex with a long axis of ~18 nm (1, 112, 224). At the tip of the “W” is the Ska1 microtubule binding domain which contains a winged helix-like domain (1), and an unstructured extension from Ska3 which mediates phospho-dependent interactions with the coiled-coils of Ndc80 complexes and enhances microtubule binding (2, 34, 94, 271). Ska complexes are able to autonomously track with the ends of depolymerising microtubules, and are able to interact with both straight and curved protofilaments (1, 94, 106, 110, 151, 174, 224). Unlike KMN, the Ska complex is largely missing from unattached kinetochores and progressively loads as microtubules bind the kinetochore (13, 36). Experiments in vivo and in vitro with purified proteins show that the Ska complex operates as a load-bearing device within the kinetochore (13, 94). This feature of Ska is reinforced by Ndc80 complexes and reduces the detachment rate from depolymerising microtubules (94).

#### DAM1 Complex

The heterodecameric budding yeast DAM1C is unrelated to Ska but performs an analogous and essential function. A single heterodecamer forms a rod-shaped complex with a near-perpendicular Spc19-Spc34 protrusion in the middle of the rod (111). Sixteen DAM1C heterodecamers make head to tail contacts at the ends of the rods to assemble into rings that encircle microtubules (111, 171, 206, 257). Each kinetochore appears to have two DAM1C rings (125). Like SKA, DAM1C association with kinetochores requires microtubules (146). The plus end-tracking protein, Bim1 (yeast EB1), binds to the DAM1C protrusion in a phospho-dependent manner, promoting its oligomerisation, and potentially handing over to Ndc80 (62). DAM1C also has similar biochemical properties to SKA, acting as a force coupler through interactions with both microtubules and Ndc80 complex (135, 136, 238).

#### Astrin-Skap Complex

In mammals, formation of a mature microtubule-kinetochore interface further involves recruitment of the microtubule-binding Astrin-Skap-MYCBP-LC8 complex, which is positioned close to the Ndc80 complex (64, 73, 121, 155, 225). Unlike KMN-S, the Astrin-SKAP is proposed to reduce friction in the kinetochore-microtubule interface (212).

In summary, KMN-SA is a key feature of kinetochores that enables coupling of chromosomes to dynamic microtubules – thus harnessing energy for powering chromosome movement. The molecular mechanisms are a combination of biased diffusion on the MT lattice (by NDC80C), binding to curved protofilaments (by SKAC) – a feature of growing and shrinking MT tips (90) or an encircling coupler (by DAM1C). Motorised tethering by kinesin and dynein motors also contributes (see section 3.4; For review and more discussion of the biophysics see: (12, 52, 149).

#### 3.3.1 KMN-SA is the major target for error correction processes

The resolution of improper microtubule kinetochore attachments involves a number of tension-dependent and independent mechanisms (For detailed discussion see (138)). Briefly, the latter “basic” mechanism depends on geometric constraints (i.e. sisters are back-to-back) and the natural turnover rate of kinetochore-microtubules. The tension-dependent mechanism is linked to the Aurora B dependent phosphorylation of key kinetochore substrates (including Ndc80, Knl1 and Ska1/Dam1). These modifications reduce the affinity of the kinetochore for microtubules promoting either release or depolymerisation (reviewed in (60, 259). We note that the SAC kinase Mps1 kinase is also implicated in promoting biorientation, in part through phosphorylation of the Ska3 hinge region and Ndc80 tail (151, 217). A key challenge is to understand how different attachment states *i.e.* amphilelic *vs* syntelic *vs* merotelic are coupled to changes in the phospho-state of the outer kinetochore. Spatial separation of kinase-substrates between centromere and kinetochore or between intra-kinetochore positions have both been proposed (137, 138). Aurora B is localized to centromeres/kinetochores through multiple receptors, suggesting that both types of model may be relevant (96)(30). For example, preventing survivin-based Aurora B (Ipl1) targeting in yeast (31) is compatible with tension-sensing because the C-terminal region of Ctf19 is an Ipl1-binding site (70, 77). Nevertheless, once a kinetochore forms an end-on attachment further attachment stabilisation takes place. This is due to the above-mentioned maturation of the outer kinetochore, BubR1-PP2A activity and recruitment of PP1 to Knl1 (219). This likely overwhelms kinase activity and explains why metaphase kinetochore do not detach under natural fluctuations in tension.

#### 3.3.2 Connectivity between sub-assembly II and III

To act as a force coupler that allows chromosome movement, the kinetochore must maintain connectivity between the inner and outer kinetochore. Several distinct paths of connectivity have been described and the relative importance of these differs between organisms. Details of phosphorylations and other posttranslational modifications that modulate sub-complex interactions are also beginning to emerge. However, despite remarkable insights into the organisation of individual sub-complexes, the overall architecture of a complete kinetochore and the regulatory events that permit this super-assembly have yet to be revealed. Two pathways of connectivity between the inner and outer kinetochore exist in both yeast and humans and involve disordered extensions of CenpC and CenpT that have the potential to project several tens of nanometers outwards from CCAN (Fig 2a, **point 2**):

- CenpC is bound directly by the MIS12C which in turn binds one NDC80C and a single KNL1. The CenpC interaction with MIS12C is facilitated by phosphorylation of two conserved serine residues on Dsn1 by Aurora B (3, 25, 92, 126, 204, 276). This displaces an autoinhibitory fragment, exposing a binding site on Mis12/Mtw1 for CenpC/Mif2 (58, 196). In yeast, Mis12/Mtw1 also binds Ame1 (104) but the reciprocal “third” CenpU-Mis12 linkage has not yet been shown in humans. An autoinhibitory mechanism similarly prevents CenpC that is not bound to the centromeric nucleosome from binding to MIS12C (123). In yeast, Aurora B may further stabilise the kinetochore through phosphorylation of CenpA (23).
- CCAN subunit CenpT can bind directly to two NDC80C (58, 79, 154, 159, 179, 189, 196, 204, 223, 226). Both MIS12C and CenpT bind NDC80C through the same interaction surface in the RWD motifs of Spc24-Spc25 (58, 102, 154, 179, 223). CenpT can also recruit one MIS12C which, in turn, brings an additional NDC80C (107).

In sum, each CCAN has the potential to recruit 5 NDC80C, 3 MIS12C and 2 KNL1 with CenpC and CenpT providing independent links to the outer kinetochore (115, 128, 163, 234). It remains unknown whether these different subpopulations of Ndc80 have differential functions or mechanical properties. These connectors are flexible (Roscioli et al, 2020) and likely operate as a compliant linkage between CCAN and KMN-SA that can withstand hundreds of piconewtons of force when microtubules are driving chromosome movement (234, 267) (Fig 2a)).

### 3.4 Sub-assembly IV (Corona – a metazoan specialisation)

The corona is the outermost layer of the kinetochores and was originally identified in electron micrographs as a diffuse fibrous network that appears when microtubules are not engaged with the kinetochore (116). The corona is highly plastic being able to undergo a time-dependent expansion to form crescents, and ultimately a structure that can encircle the entire pair of sister chromatids at the primary constriction (for review see:(130)). Several proteins are known to be part of this expansion and include the RZZ-S, Dynein-Dynactin (DD), CenpE, CenpF, Clasp, Clip170, Mad1-Mad2, Cyclin B and Nup107-160 (Table 1).

The core of the fibrous corona is the RZZ complex which dimerises to form a head-to-tail overlapping 42-nm long dimer that recruits Spindly through an interaction with Rod’s beta-propeller (175, 191, 205). This interaction requires the farnesylation of the carboxy-terminal CAAX box which releases spindly from an auto-inhibited state (214). Spindly, in turn, recruits the Dynein-dynactin motor complex which is important for future compaction (see below). RZZ-S then drives the process of kinetochore expansion and this requires Mps1 phosphorylation of Rod (211, 214) and Zwilch (191). Early experiments showed how purified Rod-Zw10 dimers can self-assemble into filament like structures (191) although self-assembly of full RZZ complexes requires farnesylated Spindly with Mps1 acting as a catalyst (205, 214). The similarity of Rod to membrane-coating proteins (*i.e.* Clathrin and COP I/II) that can form high-order assemblies points to common mechanistic principles (45, 175).

Cells deficient of RZZ do not form a fibrous corona (by EM; (211)). However other corona proteins remain kinetochore-bound albeit without undergoing expansion (*i.e.* CenpF/CenpE/Mad1 (44, 211). Corona proteins must therefore dock through RZZ-independent mechanisms, presumably, to the outer kinetochore. CenpF is an ~3000 amino acid microtubule binding protein that contains extensive coiled-coils enabling it to physically bridge the corona and outer kinetochore, where it docks onto the kinase domain of Bub1 (18, 44, 202). Similarly, CenpE, which is a member of the Kinesin-7 family, docks through its carboxy-terminus to the kinase domain of BubR1, while the amino-terminal motor domain is projected via the extended coiled-coil region into the corona. Both BubR1-CenpE and Bub1-CenpF are assembled onto Knl1 (which does not show expansion behavior). Their common features have raised the possibility they are distantly related paralogs (44).

How the RZZ-S assembles onto sub-assembly II (outer kinetochore) is less well understood. Depletion of Zwint or Knl1 reduces – but does not abolish - the binding of RZZ to kinetochores (131, 227, 244). Consistently loss of the Knl1-dependent BubR1-CenpE or Bub1-CenpF linkages does not affect RZZ binding (14, 53, 211). Thus, there must be linkages beyond Knl1 axes, with one possibility being the reported interaction between Rod and the Ndc80 complex (35). This could be consistent with nanoscale mapping experiments that locate RZZ to the outside of, but close to, the Ndc80 amino-terminus on unexpanded kinetochores (213).

It is well established that Mad1 is recruited to kinetochores through a direct interaction with Bub1 which is located in the outer kinetochore and does not itself undergo expansion (Bub1 is the only kinetochore receptor for Mad1 in yeast (148)). The carboxy-terminus of Mad1 (close to Mad2 binding site) is located proximal to outer kinetochore Bub1, while the amino terminus is ~50nm outside. This is consistent with Mad1 bridging the outer kinetochore and the corona (213) . As kinetochores expand, Mad1-Mad2 are enriched in the corona suggesting a second population and receptor. Several lines of evidence support this idea: i) Mad1-Mad2 bind detached coronas which do not contain Knl1-Bub1 (191), ii) Rod-deficient cells (no corona) still recruit Mad1 to outer kinetochores, iii) the amino-terminus of Mad1 binds directly to Corona-associated CyclinB (4).

Overall, current data suggest that kinetochores project several highly flexible molecules beyond the outer kinetochore to form, with RZZ-S, a “proto-corona” that can operate as a nucleating centre for expansion through Mps1-triggered self-assembly of corona proteins (Fig 2b). These new self-assemblies would not necessarily connect to the outer kinetochore, thus explaining how the corona can be disassociated as a single unit from the kinetochore (191).

As end-on attachments form, the corona disassembles because the minus-end directed motor activity of DD “strips” the corona from the kinetochore. Hence the corona sets up its own destruction through recruitment of DD and its activator Spindly (80, 105, 214). This could be a passive process that initiates the moment a microtubule forms an end-on attachment. Consistent with this idea is the finding that loss of Mps1 activation is neither necessary nor sufficient to trigger corona disassembly (214). Stripping may also provide a feedback to further promote end-on attachment by relieving inhibition of NDC80C by RZZ (35) – perhaps gating the straightening of NDC80 complexes and associated loss of Mad1:Mad2 (213). However, regulation is clearly important because stripping does not fully eliminate all corona proteins from kinetochores (Mad1:Mad2 is an exception). This likely reflects the observations that some factors are needed to trigger expansion (see above) while others *e.g.* CenpF and CenpE are directly implicated in coupling kinetochores to dynamic end-on attached microtubules (89, 117, 246). CenpF (via Nde1/Ndel1/Lis1) also functions as a “dynein brake” to limit stripping of corona cargoes (14). This is important because slowing or accelerating the stripping process leads to mitotic defects (14, 80). It will be important to determine whether each corona cargo is stripped at different times/kinetics and how this is coordinated with cycles of microtubule attachment-detachment.

## 3 Structural organisation of centromeric and pericentromeric chromatin

Regional centromeres contain blocks of CenpA nucleosomes linearly interspersed with lysine 4 dimethylated H3-containing nucleosomes in which CenpA nucleosomes are at a density of just ~1:25 CenpA:H3 (20, 22, 209, 231, 243). CenpA nucleosomes are gathered at one face of the chromatin to form a kinetochore assembly platform, with H3K4 trimethylated nucleosomes residing underneath (231). Evidence from chicken neocentromeres suggests that centromeric nucleosomes are densely packed (178). Intriguing recent data showed that the kinetochore protein CenpN is capable of stacking CenpA nucleosomes, suggesting it may contribute to the higher order structure of centromeric chromatin (275). This core domain of centromeric chromatin is flanked by H3 lysine 9 trimethylated (H3K9Me3) and HP1-bound pericentromeric heterochromatin, which is highly enriched with the chromosome-organising complex, cohesin (78). In fission yeast, HP1 is required for pericentromeric cohesin enrichment, while in humans HP1 may have an indirect role (17, 129, 180, 263). The repetitive nature of centromeric chromatin has precluded a detailed picture of its architecture but super-resolution and chromatin-unfolding experiments in chicken have suggested a modular structure (209, 243), proposals include a solenoid or a boustraphedon (209, 231).

**Figure 3:**
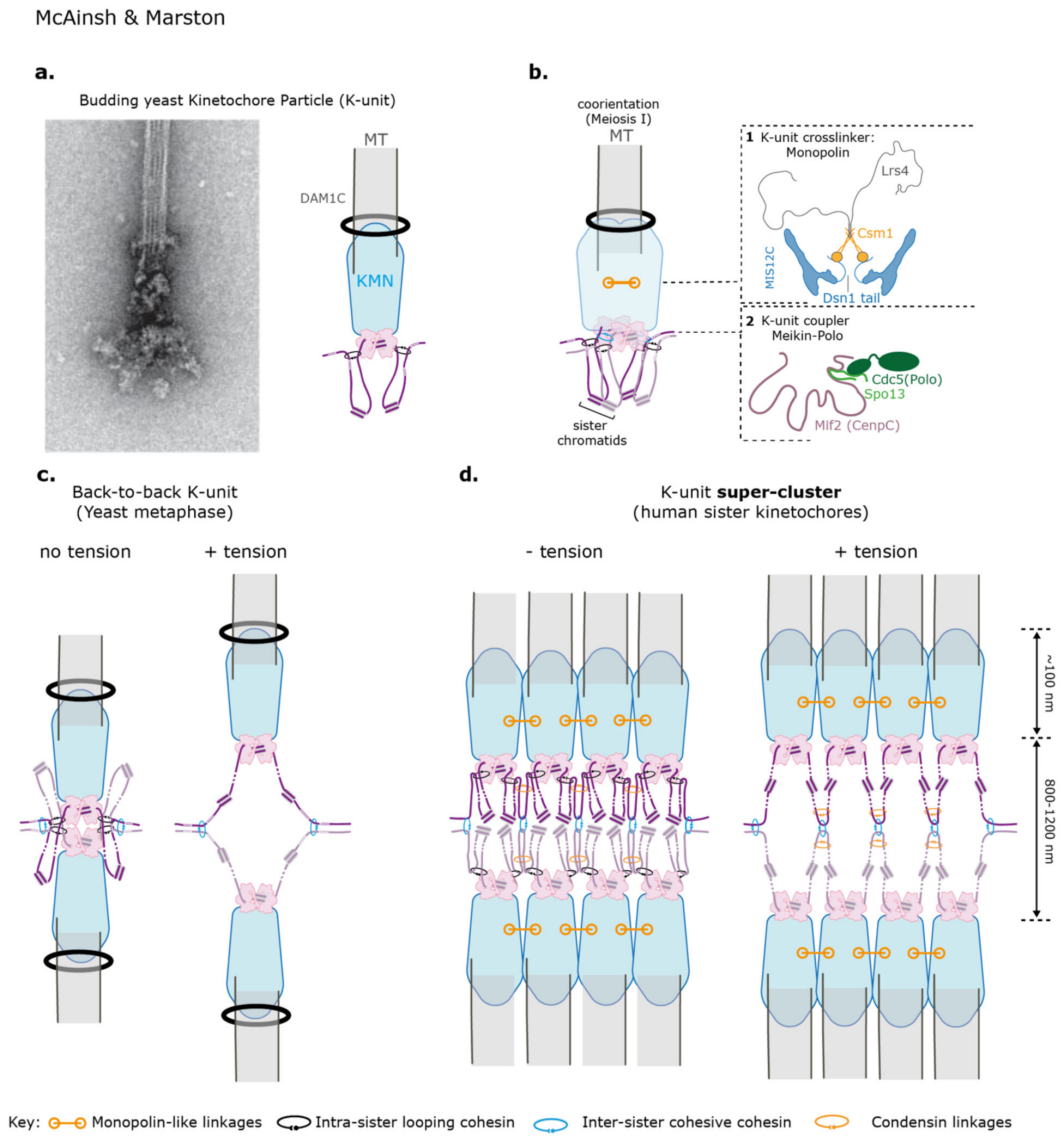
Super-cluster for centromere-kinetochore multimerisation **(a)** *left* Cryo-electron microscopy image of isolated budding yeast kinetochore particles bound to microtubules. In the image, globular domains contact the microtubule which is encircled by a ring-like structure, likely DAM1C. There is also a central hub which does not contact the microtubule directly. *Right* Model for the architecture of a single k-unit where one kinetochore superassembly contacts one microtubule, as in budding yeast. Each CCAN anchors cohesin, which forms intramolecular loops on each side of the kinetochore. **(b)** Model for sister kinetochore coorientation during meiosis I in budding yeast. Two k-units - the sister kinetochores - are clamped together in a side-by-side orientation due to two kinds of linkages. (**1**) Monopolin binds to the Dsn1 subunit of the MIS12C and fuses the sister kinetochore together. (**2**) Meikin-Polo associates with CenpC/Mif2 and promotes coorientation, possibly by facilitating cohesin-dependent-linkages of sister centromeres. Note that a fused pair of sister kinetochores binds a single microtubule in meiosis I (261). **(c)** Schematic showing the architecture of the budding yeast kinetochore in mitosis in the presence and absence of spindle tension. *Left:* CCAN-anchored cohesin extrudes a loop on either side of the centromere until blocked by convergent genes at pericentromere borders. Borders also retain inter-sister, cohesive cohesin. This state, as shown, is short-lived because the attachment of sister kinetochores to microtubules from opposite poles results in the generation of tension. *Right* Under tension, the chromatin loops extend into a V-shaped structure. Intra-molecular, loop-extruding cohesin slides off but inter-molecular, cohesive, cohesin is trapped at the borders and holds the sister chromatids together. **(d)** Speculative model for the architecture of the mammalian kinetochore, inspired by the structure of the budding yeast kinetochore and pericentromeric chromatin in mitosis and meiosis. Ordered arrays of k-units are clustered together. This clustering is facilitated by cohesin anchored on CCAN and stabilised by cross-linkers between KMN, analogus to monopolin. Chromatin-organising complexes such as condensin may further serve to stabilize interactions between adjacent K-units.

In contrast, the absence of heterochromatin and repetitive sequences has allowed the application of next generation sequencing-based approaches to probe the structure of budding yeast pericentromeres (Fig. 3a). Cohesin is enriched over ~20kb surrounding the ~125bp yeast centromere and plays a central role in pericentromere folding (66, 68, 69, 82, 186, 252). Cohesin is specifically targeted to centromeres through a direct interaction between a conserved patch on the Scc4 subunit of the Scc2-Scc4 cohesin loader and the N-terminus of the kinetochore component Ctf19 (subunit of CCAN) upon its phosphorylation by the Dbf4-dependent kinase (DDK) (100, 101). Although initial studies had suggested that the budding yeast pericentromere forms a cruciform structure (269), later Hi-C analyses found that centromeres strongly suppressed interactions between flanking chromatin on each side (51, 141, 186, 221), discounting this model. Instead, Ctf19-anchored cohesin extrudes an intra-chromosomal loop on each side of the centromere until it is stalled by convergent gene pairs which form boundaries at the pericentromere borders, which is also where cohesin links the sister chromatids (186). Upon sister kinetochore biorientation in mitosis, the loop-extruding cohesin is released from chromosomes, and pericentromeres adopt a V-shaped structure with borders at their apices ((186)) (Fig. 3c).

## 4 Meiotic kinetochores

Kinetochore adaptations during meiosis support the segregation of homologous chromosomes in meiosis I followed by sister chromatids in meiosis II (Fig. 1b) (29, 65). Following DNA replication and the establishment of sister chromatid cohesion in S phase, homologous chromosomes pair and undergo meiotic recombination. This generates crossovers which, together with sister chromatid cohesion, hold homologs together, allowing for their biorientation on the meiosis I spindle. During meiosis I, sister kinetochores are mono-oriented so that they attach to microtubules from the same pole. Homolog segregation during meiosis I is triggered by loss of arm cohesion, while pericentromeric cohesion is protected to keep sister chromatids together. During meiosis II, sister kinetochores biorient, pericentromeric cohesin is deprotected and sister chromatids segregate to opposite poles. Kinetochores play central roles in multiple aspects of meiosis, including preventing crossover recombination in pericentromeres, homolog pairing, synaptonemal complex nucleation, establishment of pericentromeric cohesin protection and sister kinetochore monoorientation (see recent reviews (28, 65, 133, 158, 182)). Here, we focus on our current understanding of how meiotic kinetochore architecture is adapted to bring about these functions.

### 4.1 Architecture of meiotic kinetochores

Budding yeast meiotic kinetochores have a similar composition to mitotic kinetochores, with the addition of meiosis-specific factors ((27, 216); see also below). However, the pathways that govern the assembly and maintenance of kinetochores may differ in meiosis compared to mitosis since kinetochore integrity and viability in budding yeast meiosis relies on CCAN subunits that are dispensable for mitotic growth (26). In the budding mitotic yeast cell cycle, kinetochores remain fully assembled except for a brief period during S phase (127). In contrast, kinetochore subassembly III (KMN-S) disassembles during meiotic prophase as a result of both reduced synthesis and increased degradation of the Ndc80 protein (11, 38, 39, 168, 170). This is reminiscent of kinetochores in mammalian somatic cells where subcomplex III (KMN-S) assembles only at mitotic entry to make kinetochores competent to bind microtubules (93). Ndc80 degradation in meiotic prophase is promoted by Aurora B kinase (Ipl1), which also severs kinetochore-microtubule attachments reminiscent of its role during error correction in mitosis (see above)(38, 168). The loss of the outer kinetochore may facilitate the remodelling of the kinetochore for meiosis. Indeed, components of the synaptonemal initiation complex and monopolin are recruited by the inner kinetochore in meiotic prophase (27). Kinetochore disassembly in meiotic prophase may also prevent centromeremicrotubule attachments at a time when telomeres are attached to microtubules to bring about the coordinated chromosome movements known as the meiotic bouquet, which is thought to facilitate homology search (222). However, preventing Ndc80 degradation in meiotic prophase does not have any obvious adverse effects on unchallenged meiosis, so the role of outer kinetochore disassembly remains unclear (38).

Mammalian meiotic kinetochores have not been intensively studied, but components of the major subassemblies appear to be present (188, 279). Furthermore, kinetochores in human oocytes are prone to fragmentation (278), raising the interesting possibility that the links between individual kinetochore assemblies (which we refer to as k-units below) become weakened over time.

### 4.2 Sister kinetochore monoorientation

The segregation of homologs, rather than sister chromatids, in meiosis I underlies Mendel’s law of segregation and requires that sister kinetochores are monooriented. Sister kinetochore monoorientation was shown to be a property of the kinetochore, rather than microtubules by pioneering transplantation experiments in grasshopper spermatocytes (187) and more recently in mouse oocytes (183). Electron microscopy in male *Drosophila* revealed that sister kinetochores orient in a side-by-side fashion and converge into a single structure in meiosis I, while light microscopy in maize indicated that sister kinetochores may be fused by a Mis12 bridge (83, 145).

In budding yeast, ultrastructural analysis of the meiosis I spindle found insufficient microtubules for each pair of sister kinetochores to have more than one microtubule (261). This, together with biophysics experiments showing that kinetochore particles isolated from budding yeast meiosis I cells are larger and can withstand higher forces than those from mitotic or meiosis II cells provides evidence for sister kinetochore fusion also in budding yeast (216).

The molecular basis of mono-orientation is poorly understood. In budding yeast, a four-subunit complex called monopolin is required for monoorientation *in vivo* and is sufficient to alter the biophysical properties of kinetochores *in vitro* (195, 203, 216, 239). Monopolin comprises two nucleolar proteins, Lrs4 and Csm1, CK1δ kinase Hrr25 and a meiosis-specific protein, Mam1. The Polo kinase, Cdc5, promotes release of Lrs4 and Csm1 from the nucleolus to form the four-protein monopolin complex at kinetochores (195, 203). Monopolin is a V-shaped complex in which Csm1 homodimers are linked at the end of their coiled-coil N termini by two Lrs4 subunits (Fig. 3b) (49, 50). The Csm1 globular heads at the apices of the V bind to a region in the N-terminus of Dsn1 which is also required for sister kinetochore mono-orientation (50, 199, 218). A flexible linker separates Mam1’s C-terminal domain which wraps around a Csm1 head and its N-terminal domain that binds CK1δ to tether it to the complex (49, 268). Monopolin is thought to fuse sister kinetochores by bridging Dsn1 molecules in sister kinetochores (49, 50, 199). A key question in this model is how does monopolin avoid cross-linking Dsn1 molecules in the same kinetochore, or homologous kinetochores? It is likely that phosphorylation controls monoorientation specificity. Indeed, two residues (S109 and S110) within the monopolin binding site on Dsn1 are phosphorylated *in vivo* and phospho-mimetic mutations increased Csm1-Dsn1 binding *in vitro*, though whether CK1δ or some other kinase is responsible remains unknown (199). Mam1 has not been identified outside budding yeast, CK1δ is widely conserved and although homologs of Csm1-Lrs4 exist in some species, they are dispensable for sister kinetochore monoorientation (85, 199). Therefore, monopolin-directed monoorientation may be a point centromere adaptation.

A group of meiosis specific kinase regulators (Mokirs), which include budding yeast Spo13, fission yeast Moa1 and mouse Meikin, appear to have a more widespread role in sister kinetochore monoorientation (76). MOKIRs are not conserved at the sequence level except at a small motif that binds Polo kinase through its Polo Binding domain (PBD) and recruits it to kinetochores. In the case of Moa1 and Meikin, kinetochore association occurs through a direct interaction with a region near the C-terminus of CenpC (Fig 3b) (24, 75, 124, 153, 160, 235). The critical role of Spo13 and Moa1 in monoorientation appears to be recruitment of Polo kinase to kinetochores (75, 150, 172). Forced kinetochore association of budding yeast Polo kinase Cdc5 to kinetochores induces monoorientation independently of monopolin, suggesting a mechanism in common with organisms that lack monopolin (75). However, retention of monopolin at kinetochores in meiosis I requires Spo13, indicating that it elicits monoorientation through both monopolin-dependent and - independent mechanisms (119, 144). Mokir-Polo substrates responsible for kinetochore monoorientation have not been identified, however budding yeast monopolin subunit Lrs4 is one likely target (160). Another attractive candidate is cohesin at core centromeres, which is required for mono-orientation in budding yeast, fission yeast and mouse, and which requires fission yeast Moa1 for its establishment (16, 176, 183, 215, 251). In further support of this idea, merged meiosis I kinetochores in mouse oocytes are individualized in anaphase I, dependent on separase activity, though whether cohesin is the relevant substrate has not been demonstrated (87, 183). Meikin is also cleaved by separase, generating a fragment that retains the ability to bind Polo and kinetochores, but with a distinct function in promoting chromosome alignment in meiosis II (153). Exactly how Mokirs and cohesin direct monoorientation and the relationship between them is an important priority for the future.

In human oocytes our understanding lags far behind. Sister kinetochores appear unfused in meiosis I and the distance between them increases with maternal age (188, 279). This is a potential cause of the high levels of aneuploidy characteristic of human oocytes, and although the underlying molecular reasons are unknown, age-related cohesin loss could be a contributing factor (86).

## 5 Working Model for the kinetochore-centromere super-cluster

The modular hierarchical architecture of the kinetochore is largely conserved, strongly suggesting that regional kinetochores may resemble repeated arrays of the budding yeast kinetochore, though the exact nature of this could take many forms. Expanding on the original idea from Brinkley (280) as extended by Musacchio to include the underlying chromatin (194), we propose molecular ideas for how mammalian/regional kinetochores are built from multiple kinetochore “units” (termed k-units from here on), where one k-unit is equivalent to the “the one kinetochore-one microtubule” yeast kinetochore (Fig. 3a). The yeast k-unit is built around a single octomeric Cse4-nucleosome (113) that wraps *CEN* DNA and associates with two CTF19C when reconstituted (256, 264). These k-units have been isolated from cells and can be visualised by electron microscopy ((84) Fig 3a). The copy number of other factors has been estimated in vivo and points to the presence of two CTF19C ~6 MIS12C and ~8 NDC80C. This agrees well with the stoichiometry of CCAN *vs* KMN assemblies (section 3.3.1):

$$2^{*}{\text{CCAN}}^{*}\to 2^{*}\text{CenpC}\left(2\;\text{NDC}80\text{C}\right)+2^{*}\text{CenpT}\left(6\;\text{NDC}80\text{C}\right)=8\;\text{NDC}80\text{C}.$$

Similar counting experiments in human cells estimated ∼244 NDC80C per kinetochore (233). Recent electron tomography now indicates there may be only ~10 microtubules per human kinetochore (122, 181, 274). This gives ~24 NDC80C per MT in humans. This hints that there are either two k-units per microtubule, or that the number of NDC80C is over estimated. The reverse calculation starting from the estimated number of nucleosomes per kinetochore (22) gives:

$$44\;\text{CenpA-nucleosomes}\to {\text{88}}^{*}\text{CCAN}\to 352^{*}\text{NDC}80=35^{*}\text{NDC80C/MT}.$$

This assumes that every CenpA nucleosome brings 2*CCAN. Given the degree of structural conservation we suggest that this is not the case and that the number of NDC80C per k-unit is ~10 with a fraction of CenpA nucleosomes in position to bind CCAN (see below). Future work using knock-in human cell lines rather than transgenes should settle this. It will then be crucial to measure molecule numbers for all other components, defining stoichiometries and building molecular-scale models of the full ensemble.

How these complexes and proteins are arranged within the k-unit is also of importance. Eyeballing the electron micrographs of yeast kinetochore particles suggests an ordered structure with what are presumably NDC80 complexes projecting outwards along the MT axis from an inner core (Fig 3a). High-resolution fluorescence microscopy experiments provide evidence that NDC80C (as well as Mad1 and RZZ) have a high nematic order (that is a measure of the degree of alignment of molecules) in human kinetochores (213). This ensemble-level view further suggests that k-units must be well-aligned. Consistently, FRET experiments show that Ndc80/Nuf2 clusters and aligns in both yeast and humans, while only in the latter do Mis12/Spc25 components cluster (134). This hints at differences that likely reflect analysis of single k-units *vs.* k-unit arrays.

Alignment and clustering of k-units into a single uni-directional microtubule-binding surface would require that individual k-units are clustered together in a side-by-side manner to form a compound kinetochore (Fig. 3d). We suggest that two types of linkages couple adjacent k units: (i) protein-protein bridges juxtaposing KMN assemblies in adjacent k-units and (ii) topological chromatin organisers that link loops anchored at each k-unit. We speculate about the molecular nature of both types of linkages, taking inspiration from yeast pericentromeres and meiotic kinetochores, respectively.

### 5.1 Inter-k-unit protein crosslinkers

An innovation that is required at regional centromeres but not point centromeres is the ability of k-units on each chromosome to act in unison and form a single, complex kinetochore. We invoke a requirement for k-unit to k-unit crosslinkers in the formation of a “compound” kinetochore. Such cross-linkers are already known to function in budding yeast meiosis, where the monopolin complex bridges two microtubule binding sites representing the point sister kinetochores, through a direct interaction with the MIS12C subunit, Dsn1 (49, 50, 199, 216). Similarly, Pcs1-Mds4, which are the fission yeast orthologs of monopolin subunits Csm1-Lrs4, clamps microtubule binding sites together in mitosis to prevent merotely - attachment of a kinetochore to microtubules form opposite poles (85). Therefore, an attractive idea is that monopolin also links k-units in the compound mammalian kinetochore by bridging their KMN assemblies. However, monopolin orthologs have not been identified in metazoans, though they are found in some plants (199), and factors that link k-units have not been described. This suggests that as-yet-unidentified proteins might perform this function to stabilize the compound kinetochore.

### 5.2 Inter k-unit chromatin linkages

The ensemble emerging from multiple k-units and hundreds of proteins is not static and should not be thought of as “ribosome-like”: the shape of the kinetochore is heterogeneous, can be deformed along its microtubule axis with the outer kinetochore capable of swivelling/tilting relative to the inner – all aspects responding to changes in microtubule binding and/or force (152, 207, 213, 229, 249). This likely reflects a degree of spacing/flexibility between k-units. At the same time, there must be sufficient stiffness to withstand force and k-units should presumably be co-oriented.

Inspired by the structure of the yeast pericentromere where cohesin organises separate chromatin loops on each side of the centromere, we suggest that each k-unit may adopt a bilateral loop structure which would facilitate the coalescence of k units into a complex kinetochore. Interestingly, CenpU harbors a cohesin-binding motif (147) raising the possibility that human CCAN anchors loop-extruding cohesin similar to yeast Ctf19C, albeit through a different CCAN subunit (Table 1) (100, 186). Reminiscent of the convergent genes at pericentromere borders in yeast (186), human k-units may be flanked by transcriptional units that act as boundaries to halt loop extrusion. There is abundant evidence for non-coding transcription in centromeres supporting this possibility (143). Compound kinetochores may also require stabilization via intra-molecular linkages between chromatin loops, or between CenpA chromatin blocks. The condensin complex may contribute to this function since it is required for centromere rigidity and kinetochore geometry in both yeast and humans (208, 230, 245).

### 5.3 Other models

Our “super-cluster k-unit” model for the mammalian kinetochore is broadly compatible with previous proposals. Hill’s “sleeve” model asserts that individual microtubules insert into channels on the outer surface of the kinetochore to form multiple low affinity binding sites (95). In our model, each k-unit would be equivalent to a single channel. The “fibrous network” model advocates that a flexible meshwork of NDC80 complexes on the surface of the outer kinetochore embed microtubule ends (59). Zaytsev and colleagues argue that NDC80 complexes have low cooperativity and make multiple low affinity and independent interactions with microtubules, rather than acting as part of an oligomeric assembly (270). The clustered k-units we propose could result in overlapping NDC80 extensions to form such a meshwork and be compatible with independent NDC80 binding, consistent with both of these models.

## 8 Conclusions

The past few years have seen major advances in our understanding of kinetochore biology. The molecular structure of the majority of individual sub-complexes has emerged and we now have a working model for how they connect together into a microtubule-binding super-cluster. Questions of how force is generated at kinetochores and coupled to chromosome movement have begun to be addressed owing to developments in technology that have allowed physical properties to be measured although making such measurements in living cells remains a major challenge, with exciting recent progress in this direction (232). Kinetochores have also been revealed to influence and organise the surrounding chromatid, establishing them as much more than machines that couple chromosomes to microtubules. Indeed, *in situ* cryo-electron tomography now shows how the human kinetochore is sitting within a centromeric chromatin pocket with expected filament-like linkages to microtubules visible (274).

In this review, we have focused on the best understood kinetochores – those of budding yeast and humans, emphasising their similarities and differences (Table 1; see also Fig. 1a which highlights the dramatic difference in scale from the spindle-level viewpoint). Nevertheless, the structural organisation of yeast and human kinetochore subcomplexes is remarkably conserved, leading us to propose a modular k-unit repeat structure for the human kinetochore, based on the simpler yeast kinetochore. The deviations in complexity presumably reflect the need to up-scale the kinetochore in humans. Such up-scaling allows the attachment of multiple microtubules to a single chromosome which together form a k-fiber, providing the resilience for movement of larger chromosomes over longer distances. The multi-microtubule compound kinetochore also signifies a change in force couplers from a ring around the microtubule (DAM1C; yeast) to a system involving coupling Ndc80 to the lateral surface of microtubules (Ska; humans). A further innovation in human, but not yeast, kinetochores is the Corona, which can undergo considerable expansion. In human cells, but not yeast, the nuclear membrane breaks down at mitotic entry, spilling chromosomes into the cytoplasm and posing a significant challenge for kinetochore capture by microtubules at metaphase. Potentially the corona evolved to meet this challenge by providing a larger surface area both for kinetochore capture and to propagate the “wait anaphase” signal in response to unattached kinetochores.

Building on this foundational work, the next frontiers in kinetochore research are to reveal the ultrastructure and dynamics of the kinetochore *in vivo*. Atomic resolution cell biology will uncover how kinetochores bind microtubules and how different microtubule binding sites are coordinated within a single kinetochore. To dissect mechanisms underlying canonical and non-canonical functions of kinetochores, structure-guided designer mutations are needed to disrupt key interfaces, preclude post-translational modifications or prevent enzyme docking. Much is also to be learnt from studying the diversity of kinetochores. This includes organisms that use a variation on the theme of yeast and human kinetochores discussed here *e.g.* CCAN-lacking fruit flies and holocentric worms, and those with a completely distinct blueprint for kinetochores, such as non-canonical trypanosomes and dinoflagellates where the kinetochore remains embedded in the nuclear envelope (61). Kinetochore proteins can even play roles away from the chromosome having been shown to interact with cytoplasmic microtubules to direct neuronal development (reviewed in (57)). Only through analysis of kinetochores and their constituent parts in these distinct contexts will we gain a holistic view of kinetochore assembly and function.

## Figures and Tables

**Table 1:** Parts list of budding yeast and human kinetochores and centromeres

Protein/component		Sub-complex	Complex	Sub-	Notes
H. sapiens	S. cerevisiae			assembly	
CenpA	Cse4	-	Nucleosome	I	Non-specific DNA binding (wraps AT- rich CDEII in *Sc*)
Mis18α		MIS18C	MIS18-HJURP	I	CenpA loading machinery
Mis18β		MIS18C	MIS18-HJURP	I	CenpA loading machinery
Mis18bp		MIS18C	MIS18-HJURP	I	CenpA loading machinery
HJURP	Scm3	-	MIS18-HJURP		CenpA chaperone (binds CenpA:H4)
Shugoshin-1	Sgo1^	-	-	-	Cohesion protection; PP2A receptor
Shugoshin-2	Sgo1^	-	-	-	Cohesion protection; PP2A receptor
CenpB	n/a	-	n/a	I	DNA binding (CenpB box)
n/a	Cbf1	-	n/a		DNA binding (CDEI)
n/a	Ndc10	-	CBF3	I	DNA binding (sequence independent)
n/a	Ctf13	CBF3core	CBF3	I	DNA binding (CDEIII)
n/a	Cep3	CBF3core	CBF3	I	DNA binding (CCG motif in CDEIII)
n/a	Skp1	CBF3core	CBF3	I	F-box protein
CenpC	Mif2	-	CCAN/Ctf19C	II	DNA binding (AT hook); Dimer
CenpH	McmI6	HIKM	CCAN/Ctf19C	II	
CenpI	Ctf3	HIKM	CCAN/Ctf19C	II	
CenpK	Mcm22	HIKM	CCAN/Ctf19C	II	
CenpL	Iml3	NL	CCAN/Ctf19C	II	
CenpM	McmI6	HIKM	CCAN/Ctf19C	II	
CenpN	Chl4	NL	CCAN/Ctf19C	II	
CenpO	Mcm21	OPQUR/COMA	CCAN/Ctf19C	II	
CenpP	Ctf19	OPQUR/COMA	CCAN/Ctf19C	II	
CenpQ	Okp1	OPQUR/COMA	CCAN/Ctf19C	II	Ndc80-like MT binding
CenpR	n/a	OPQUR	CCAN/Ctf19C	II	
CenpS	Mhf1*	SX	TWSX	II	Histone-fold; also DNA repair role
CenpT	Cnn1	TW	TWSX	II	Histone-fold
CenpU	Ame1	OPQUR/COMA	CCAN/Ctf19C	II	Receptor for Plk1 (*Hs*)
CenpV	n/a	-	-		GFA domain, *CEN* chromatin structure; meiosis, MT binding
CenpW	Wip1	TW	TWSX	II	Histone-fold
CenpX	Mfh2*	SX	TWSX	II	Histone-fold; also DNA repair role
n/a	Nkp1	Nkp1/2	CCAN/Ctf19C	II	
n/a	Nkp2	Nkp1/2	CCAN/Ctf19C	II	
Spc24	Spc24	NDC80	KMN-S	III	RWD domains
Spc25	Spc25	NDC80	KMN-S	III	RWD domains
Ndc80	Ndc80	NDC80	KMN-S	III	CH domain/MT lattice binding
Nuf1	Nuf2	NDC80	KMN-S	III	CH domain/MT lattice binding
Knl1	Spc105	KNL1	KMN-S	III	MELT array as platform for SAC; PP1 receptor and MT binding (N-term)
Zwint	Kre28	KNL1	KMN-S	III	
Mis12	Mtw1	MIS12	KMN-S	III	
Nnf1 (Pmf1)	Nnf1	MIS12	KMN-S	III	
Nsl1	Nsl1	MIS12	KMN-S	III	
Dsn1	Dsn1	MIS12	KMN-S	III	Receptor for monopolin
Ska1	n/a	SKA	KMN-S	III	Load bearing device; MT Tip tracking -
Ska2	n/a	SKA	KMN-S	III	binding curved protofilaments
Ska3	n/a	SKA	KMN-S	III	
Cdt1	Tah11*			III	Also a replication factor; can bind Ndc80 loop
Ch-TOG	Stu2	-	-	III	MT polymerase; docks Ndc80 4-way junction
n/a	Dam1	-	Dam1C	III	MT Encircling-coupler
n/a	Duo1	-	Dam1C	III	
n/a	Dad1	-	Dam1C	III	
n/a	Dad2	-	Dam1C	III	
n/a	Dad3	-	Dam1C	III	
n/a	Dad4	-	Dam1C	III	
n/a	Spc34	-	Dam1C	III	
n/a	Spc19	-	Dam1C	III	
n/a	Ask1	-	Dam1C	III	
n/a	Hsk3	-	Dam1C	III	
Astrin	n/a	-	Astrin/Skap	III	
Skap	n/a	-	Astrin/Skap	III	
MYCBP	n/a	-	Astrin/Skap	III	
LC8	n/a	-	Astrin/Skap	III	
Bub1	Bub1	BUB1-BUB3	SAC	III	Protein kinase
Bub3	Bub3	BUB1-BUB3	SAC	III	Phospho-MELT binding
BubR1	Mad3	-	SAC	III	Mad3 lacking pseudo-kinase domain found in BubR1
Mad1	Mad1	MAD1-MAD2	SAC	III (& VI)	Forms mitotic checkpoint complex
Mad2	Mad2	MAD1-MAD2	SAC	III (& VI)	(MCC) with BubR1 and Cdc20.
Mps1	Mps1	-	SAC	III	Protein kinase (MELTs, xxx)
p31 comet	n/a		SAC		SAC inhibitor
CenpE	n/a	-	Corona	IV	Kinesin-7 MT plus-directed molecular motor
CenpF	Slk19	FNNL	Corona	IV	Homology unclear; MT binding and DD regulator in *Hs*.
Rod	Sec39 and Sec31*	RZZ-S	Corona	IV	Required for vesicle tethering in yeast
Zw10	Dsl1* and Tip20	RZZ-S	Corona	IV	Required for vesicle tethering in yeast
Zwilch	n/a	RZZ-S	Corona	IV	
Spindly	n/a	RZZ-S	Corona	IV	Dynein adapter
-	-	Cytoplasmic Dynein*	DD; Corona	IV	Minus-directed molecular motor (Dynein Heavy chain + 5 light/intermediate chains)
-	-	Dynactin*	DD; Corona	IV	Dynein cofactor (11 subunits)
Lis1	Pac1*	FNNL	Corona	IV	Dynein cofactor
Nde1	Ndl1*	FNNL	Corona	IV	Dynein cofactor
Ndel1	Ndl1*	FNNL	Corona	IV	Dynein cofactor
Clasp	Stu1	-	Corona	IV	MT rescue factor
Nup107	Nup84*	NUP107-160 (NPC-Y)	Corona	IV	Also a nuclear pore component
Nup133	Nup133*	NUP107-160 (NPC-Y)	Corona	IV	Also a nuclear pore component
Nup96	Nup145C*	NUP107-160 (NPC-Y)	Corona	IV	Also a nuclear pore component
Sec13	Seh1*	NUP107-160 (NPC-Y)	Corona	IV	Also a nuclear pore component
Nup160	Nup120*	NUP107-160 (NPC-Y)	Corona	IV	Also a nuclear pore component
EB1	Bim1	-	-	-	MT end-tracker
HSET†	Kar3	-	-	-	Kinesin-14 MT minus-end directed molecular motor
Kif18a	Kip3	-	-		Kinesin-8 MT plus-end directed molecular motor and depolymerase; located on KT proximal K-fibre
-	Csm1	-	Monopolin	-	Also in the nucleolus
-	Lrs4	-	Monopolin	-	Also in the nucleolus
-	Mam1	-	Monopolin	-	Meiosis-specific
CSKN1D†	Hrr25	-	Monopolin	-	Protein kinase CK1d
Meikin	Spo13	-	-	-	Meiosis-specific kinase regulaltors (MOKIRs)
AuroraB	Ipl1	-	CPC	-	Protein kinase
Survivin	Bir1	-	CPC	-	CPC localisation
Borealin	Nbl1	-	CPC	-	CPC localisation
INCENP	Sli15	-	CPC	-	Kinase activation (IN-box)
MCAK	n/a	-	-	-	Kinesin-13 MT catastrophe factor
PP1	Glc7	-	-	-	Protein phosphatase 1
PP2A-B56	PP2A-Rts1	-	-	-	Protein phosphatase 2A
Plk1 (Polo)	Cdc5	-	-	-	Protein kinase
Haspin	Hsk1*/2*	-	-	-	Protein kinase (H3T3ph)
Cyclin B		Cyclin B-Cdk1	Corona	IV	Binds Mad1
Bod1	-	-	-	-	Regulatory subunit for PP2A
SENP family	Ulp2				Docks to Ctf3/CenpI; desumolase

Abbreviations: **MT** (Microtubule); **n/a** (no confirmed orthologue);

no evidence for kinetochore localisation in budding yeast* or humans**†**;

^ Budding yeast has a single shugoshin protein that has functions in common with mammalian Sgo1 and Sgo2.
